# Sex Differences in Intergenerational Income Transmission and Educational Attainment: Testing the Trivers-Willard Hypothesis

**DOI:** 10.3389/fpsyg.2017.01879

**Published:** 2017-11-07

**Authors:** Katharina E. Pink, Anna Schaman, Martin Fieder

**Affiliations:** ^1^Department of Anthropology, Faculty of Life Sciences, University of Vienna, Vienna, Austria; ^2^Family and Population Studies, Centre for Sociological Research, KU Leuven, Leuven, Belgium

**Keywords:** socioeconomic status, parental resources, income, educational attainment, Trivers-Willard hypothesis, sex differences

## Abstract

From an evolutionary point of view, sex differences in intergenerational transmission of income may be influenced by the Trivers-Willard (T-W) effect: Low status parents should invest more in daughters, whereas high status parents are expected to invest more in sons. This bias in parental investment may result in status-dependent sex biased parental support for higher education and educational attainment and should therefore affect the level of intergenerational income transmission for the sons and daughters. We used the data from the Wisconsin Longitudinal Study (WLS) to model the effect of parental financial investment on the child's income and educational attainment controlling for the number of siblings. The observed sex differences in intergenerational income transmission demonstrate that sons profited more from parental income and education in terms of their own income than daughters. Furthermore, we showed that fathers with a high socioeconomic index (SEI) invest more in their sons' education in terms of completed years of education and financial support during college. In contrast daughters of low SEI fathers completed more years of education and received more financial support than sons of low SEI fathers. However, the pattern in intergenerational income transmission might be better explained as a product of sociological factors and reproductive trade-offs in later life rather than as a consequence of the T-W effect.

## 1. Introduction

In modern western societies, access to resources is mainly determined by wealth that in turn is generated by income and to some degree by inheritance. Parental resources contribute to children's wealth in a variety of ways: Not only do parents leave substantial sums to their offspring upon their death, but they actively invest in their children's education and career opportunities which in turn may increase their offspring's future income. However, daughters and sons may not profit equally from their parents' resources (Smith et al., 1987; Cox, 2003).

From an evolutionary point of view, men's striving for wealth and status can be explained by the association between their access to resources and higher reproductive success. The effect of status on male reproductive success has been demonstrated both in modern and historical populations. Men with high social status father more children in modern western societies (Hopcroft, 2006, 2015; Nettle and Pollet, 2008) and in hunter-gatherer societies samples (Smith et al., 2010). By contrast, women's income and occupational attainment negatively affect reproductive success (Fieder and Huber, 2007, 2012). Parents' incomes not only influence the number of children, but also their children's quality in terms of status, which in turn affects the number and quality of their grandchildren. Trivers and Willard (1973) predicted that parental investment should be directed in a larger proportion to the subset of their offspring that are likely to produce more offspring in turn. Low status males are less likely to reproduce than low status females, while high status males are expected to produce more offspring than high status females (Hopcroft, 2006, 2015; Fieder and Huber, 2007, 2012; Nettle and Pollet, 2008). Therefore, it can be expected that low status parents direct their resources toward their female children, whereas high status parents should favor male children. While the Trivers-Willard (T-W) effect has been demonstrated in many species (for a review see Cameron, 2004), evidence in human societies is inconclusive (Lazarus, 2002). Dickemann (1979), Boone (1986), Voland et al. (1997) found such effects in data from historical India, China and Europe: Low class families tended to favor daughters more than upper class families. Cronk (1989) showed among the low status Mukogodo in East Africa that parents biased their investment in favor of their daughters. The results are less consistent in contemporary industrial societies: Hopcroft (2005) showed that daughters of high status Americans attain a lower educational status than sons, whereas daughters of low status Americans attain a higher educational status than sons. Koziel and Ulijaszek (2001) showed in a Polish sample that sons with higher educated fathers were breastfed for a longer time than girls. Keller et al. (2001) found no differential parental investment in the time per week spent with sons and daughters respectively, nor in measures of breastfeeding and self-perceived relationship quality. Hopcroft (2005) argues that these conflicting findings may be the results of self-reported proximate measures of paternal investment, since parents would have to admit to favoring one child. She further argues that wealthy societies may not have large sex differences in these certain kinds of investment at the early stage of childhood, therefore parental support for educational attainment may be a more appropriate measure to investigate a status-dependent sex bias in parental investment.

Many studies in sociology have demonstrated the importance of parental investment on offspring's status attainment (e.g., Blau and Duncan, 1967; Sewell and Shah, 1967; Sewell and Hauser, 1975; Hauser and Featherman, 1977; Sirin, 2005). Few of them have focused on the effect sizes of these factors (e.g., Hauser et al., 1973; Solon, 1992). All of these studies have focused on the interaction between a father's education and occupational attainment on the son's education, occupational attainment and income, but disregarded the effect of the mother's socioeconomic status and the influence of parental status on daughters. Therefore, it is difficult to judge to which extent parental status can be considered a relevant influence on the offspring's position in society and whether a T-W effect exists in a population. In order to compare the benefit from parental education vs. parental income for both sexes, this study focuses on quantifying the effects in easily interpretable units.

The association between parental income and children's incomes is often expressed as an intergenerational income elasticity (Becker, 1986). Intergenerational income elasticity describes the percentage of the parental income deviation from the mean that is transmitted to their children. For instance, at an intergenerational income elasticity of 50% children whose parents earn $1,000 more than the average for their country are expected to earn $500 more than average (Becker, 1986). For modern western societies, this measure varies between 0.115 and 0.662 for sons (Solon, 1992; Mayer and Lopoo, 2005). Because of women's traditionally limited access to the labor market, the effects of parental income on daughters' income are often disregarded with the notable exceptions of Minicozzi (1997), Chadwick and Solon (2002) and Shea (2000). These studies reported an intergenerational income elasticity between 0.39 and 0.41 (Minicozzi, 1997; Shea, 2000) for daughters. In general, parental income is a weaker predictor of future annual income for daughters than for sons. Hauser et al. (1973) used a path model to estimate the effects of the father's income and education on the son's income for the Wisconsin Longitudinal Study (WLS) dataset. He concludes that the effects of the father's education, occupational attainment and other socioeconomic background variables are negligible when correcting for their correlation with paternal income.

In addition to parental income and education, the number of siblings may affect the amount of resources that parents invest in each child (Butcher and Case, 1994; Sulloway, 2001; Black et al., 2005). Lawson and Mace (2009) showed that a larger number of children lead to a lower frequency of interactions between each parent and child. Low parental involvement is associated with high rates of high school dropout and low educational attainment (e.g., Fan and Chen, 2001; Barnard, 2004; Hill and Tyson, 2009). A higher number of siblings may therefore negatively affect a child's income.

In contrast to other studies this study aimed to test the T-W hypothesis in a sample where respondents had already completed their reproductive lives. Furthermore, we compared children's yearly income with their parents' yearly income at a point in time where both of them had similar ages. We tested the T-W hypothesis by using children's yearly income, their educational attainment and their parents' ability to financially support them in college as a proxy for non-biological parental investment. We aimed to quantify the effects of parental income on the next generation's income for the WLS data set. Both parents' education and their pooled income were used to predict their children's income. We controlled for the number of siblings and compared the magnitude of the above-mentioned effects for sons and daughters.

Some scholars argue that the socioeconomic index (SEI) is a more appropriate measure for testing the T-W hypothesis than parental income and parental education (Hopcroft, 2005; Hopcroft and Martin, 2014, 2016). Therefore we tried to replicate the findings of Hopcroft and Martin (2014, 2016) with the WLS. We used SEI of fathers to predict their children's educational attainment. As an additional measure of parental investment, we compared the extent to which parents supported their sons and daughters to go to college.

We expected both parental education and parental income to augment children's income. Based on the T-W hypothesis we predicted that sons profit more from parental resources in terms of their income than daughters. We expected the sex difference in educational attainment to differ for sons and daughters of fathers with different SEI. We predict daughters of low SEI fathers to have a higher educational attainment relative to sons in comparison to daughters of high SEI fathers. In addition, we expected daughters of low SEI fathers to be more supported in terms of their college education relative to sons when compared to daughters of high SEI fathers.

## 2. Materials and methods

WLS (http://www.ssc.wisc.edu/wlsresearch/) goes back to the state-sponsored questionnaire administered in 1957 to all students in all Wisconsin high schools in their final high school year. One third of this original cohort was randomly selected for further data collection. This random sample contains data from 10,317 Wisconsin high school graduates (5,326 women and 4,991 men) born between 1937 and 1940.

In 1957 and in 1964 information about the respondents' parents was collected such as *parental income* in $100 and the *parental education*, which comprises the *father's education* in years and *the mother's education* in years. *Parental income* was collected from the Wisconsin state income tax records for 1957–1960 and includes income generated by the mother and the father. The father's mean age at that time was 50 years (SD ± 7 years) and mother's mean age was 46 years (SD ± 6 years). At that time, respondents were between 19 and 21 years old (mean age: 18 years, SD ± 0.5 years). To measure familys social status we used *father's SEI* when respondents were in their final high school year, coded using Duncan SEI scores (Blau and Duncan, 1967). The value from the Duncan SEI score ranged from 1.00 to 96.00 in the WLS. Respondents were asked to state their sex coded male = 1, female = 0. In addition, respondents were asked to state their *total number of siblings ever born*. In the 1992/1993 follow-up, the respondents were asked to report their most recent *income* in US dollars before deductions. Income was measured when the respondents were between 52 and 55 years old (mean age 53 years, SD ± 0.5 years). We selected this time period instead of an earlier one where respondents were between 35 and 38 years (mean age 36 years, SD ± 0.5 years) because at this point women's reproductive periods are over, allowing them to return to the labor market. In addition, at this point in live, the income has already reached a stable level (Becker, 1994) and the age of respondents (mean age 53 years, SD ± 0.5 years) is comparable to the mean age of their parents at the time the parental income was measured. In our analysis, we included only respondents who reported a non-zero income. We did not differentiate between part-time and full-time workers. As an additional proxy for parental investment we used respondent's reported *education* in years. Furthermore, we used the variable *parents are able to support college* measured in three categories (cannot support college = 1, can support college with scarifies = 2 and can support college easily = 3).

### 2.1. Statistical analyses

Since the distributions of *respondents' income* and *parental income* are skewed, we performed a square-root transformation of both parameters before computing the regression models. Based on the descriptive analysis of the dataset, we designed a model woman and a model man for whom we then could compare the effects of parental status predicted by our regression model.

To model income, we started with a linear regression model predicting the square-root of the *respondent's income* from the *total number of siblings*, the square-root of the *parental income, mother's education, father's education* and the *respondent's sex* as well as all interactions between sex and the remaining independent variables. Because of multicollinearity, we could not include birth order in our models as it is highly correlated with the number of siblings (*r* = 0.525, *N* = 9,524, *p* < 0.001). For the same reasons we could not use the number of brothers (*r* = 0.756, *N* = 9,526, *p* < 0.001) and the number of sisters (*r* = 0.744, *N* = 9,526, *p* < 0.001). In a stepwise algorithm with the AIC as a criterion, we eliminated all irrelevant interactions. To evaluate the robustness of our model's coefficients, we repeated our analysis omitting one of the independent variables (and, if applicable, the interaction between the variable and the respondent's sex) at a time. We calculated the derivatives of the square of the income model to be able to quantify the effects of the independent variables in terms of dollars earned per year. Due to the fact that intergenerational income transmission favors sons over daughters we used educational attainment and parents' ability to support college as a proxy of parental investment. We started with a linear regression model predicting the *educational attainment* from *total number of siblings*, the *father's SEI* and the *respondent's sex* as well as all interactions between sex and the remaining independent variables. We used a stepwise algorithm with the AIC as a criterion to eliminate all irrelevant interactions. To model parental ability to support college we used a ordered logistic regression model predicting *parents' ability to financially support child in college* from *total number of siblings*, the *father's SEI*, and the *respondent's sex*. In the two models where we used *father's SEI* as a proxy for parental status we didn't include *father's education* and *mother's education* due to the fact that *father's education* is incorporated in the variable *father's SEI*. Statistical analyses were carried out with R version 3.2.2 (R Core Team, 2013) and SPSS 24.

## 3. Results

### 3.1. Descriptives

Measures of central tendency and variance of distribution for all variables used in the empirical analysis are reported in Table 1. For the comparison of the effects of early life factors on women and men, respectively, we examined a model woman and a model man: Each has three siblings (the rounded mean number of siblings), parents who earned the median income for this sample, a father with ten years of schooling (rounded mean) and a mother with eleven years of schooling (rounded mean).

**Table 1:** Descriptive statistics: model women and model men.

**Variable**	**Women**	**Men**	**All**
Median parental income in $	5,400	5,400	5,400
(Q1)	(3,400)	(3,400)	(3,400)
(Q3)	(7,400)	(7,400)	(7,400)
Mean father's SEI	30.5	30.4	30.4
(S.D.)	(22.1)	(22.2)	(22.1)
Median respondent's income in $	18,000	41,000	30,000
(Q1)	(10,000)	(30,000)	(16,000)
(Q3)	(30,000)	(60,000)	(45,000)
Mean mother's education (yrs)	10.5	10.7	10.6
(S.D.)	(2.9)	(2.9)	(2.9)
Mean father's education (yrs)	10.2	10.4	10.3
(S.D.)	(3.1)	(3.2)	(3.1)
Mean respondent's education (yrs)	13.2	13.8	13.5
(S.D.)	(1.8)	(2.2)	(2.1)
Mean number of siblings	3.3	3.2	3.2
(S.D.)	(2.6)	(2.5)	(2.6)

### 3.2. Effects of parents' status on their children's income

#### 3.2.1. Model coefficients

As expected, both the father's and the mother's education contribute significantly to their child's income (Table 2). However, the size of the effect depends on the child's sex. An additional year of education to the mean duration of the father's education benefits a son by additional $873.73 per year. Compared to this, a daughter gains only $156.62 to her yearly income. In contrast, an additional year of the mother's education (12 instead of the mean number of 11 years of schooling) benefits a son's income by additional $1,008.04 per year, whereas a daughter's yearly income increases by $368.98 (Table 2).

**Table 2:** Effects of parental income† on respondent's income†.

**Variable**	**Est. of Coefficient**	**Std. Error**	***t*-value**	***p*-value**
Intercept	108.783	6.134	17.733	<0.001^***^
Parental income†	1.066	0.536	1.989	0.047^*^
Father's education (yrs)	0.576	0.490	1.175	0.240
Mother's education (yrs)	1.358	0.494	2.748	0.006^**^
No. of siblings	−0.445	0.387	−1.162	0.245
Sex (male = 1)	28.097	8.113	3.463	<0.001^***^
Sex^*^Father's education (yrs)	1.753	0.694	2.526	0.011^*^
Sex^*^Mother's education (yrs)	1.330	0.719	1.850	0.064
Sex^*^Parental income†	2.6487	0.740	3.581	<0.001^***^

† *Variable is square-root transformed*.

* p < 0.05;

** p < 0.01;

*** *p < 0.001*.

**Figure 1:**
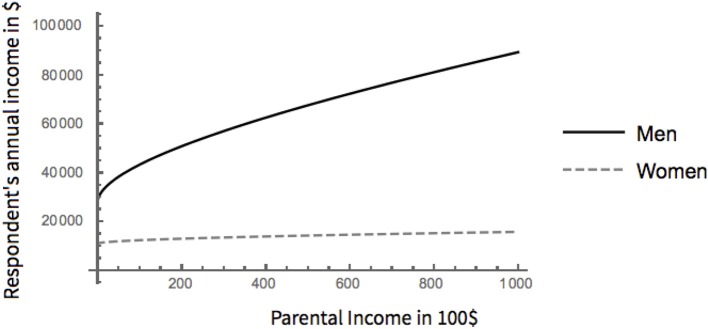
Effect of parental income on respondent's income for men and women.

In accordance with our prediction, the child's sex not only affects his or her income, but also the extent to which this income is determined by parental status (Figure 1). Not only is women's income lower in this sample, women also benefit less from their parents' income in terms of their own income. If only respondents with a regular income are considered, women's median income is $18,000 per year compared to men's median income of $41,000. Taking into account the effects of parental income and of the interactions with sex, for each extra 100 dollars parents of a median income earn per year, a model daughter gains $19.72 of additional income per year. In contrast, a comparable model son benefits by additional $94.82 per year.

The number of siblings does not affect the respondent's income significantly in our main model. However, if either the parental income or the mother's education is omitted as a predictor, the number of siblings has a significant, yet only a small negative effect on the respondent's income.

#### 3.2.2. Fit and robustness of coefficients

The income model explains 29% of the observed variance in respondent's income. As there is some degree of collinearity in our predictors *father's education* and *mother's education* are correlated with *r* = 0.491 (Spearman correlation, *N* = 9,228, *p* < 0.001), *parental income* is correlated with *r* = 0.347 to *father's education* (Spearman correlation, *N* = 8,296, *p* < 0.001), we repeated the analysis omitting one of our independent variables at a time to observe the change in the estimate of coefficients (see Table 3).

**Table 3:** Effects of omitting independent variables on our income† model.

**Without**	**Ic**	**Sib**	**Ef**	**Em**	**pI**	**Sex**	**SEf**	**SEm**	**SpI**	**Fit**
Sibs	106.164	−	0.619	1.388	1.122	28.340	1.774	1.336	2.643	0.298
	(5.706)	−	(0.489)	(0.493)	(0.534)	(8.111)	(0.693)	(0.719)	(0.740)	
	^***^			^**^	^*^	^***^	^*^		^***^	
Ef	111.402	−0.557	−	1.577	1.236	33.339	−	2.243	3.091	0.295
	(5.848)	(0.387)	−	(0.456)	(0.514)	(7.790)	−	(0.650)	(0.712)	
	^***^		−	^***^	^*^	^***^	−	^***^	^***^	
Em	117.337	−0.624	1.088	–	1.226	34.935	2.296	–	2.748	0.294
	(5.432)	(0.387)	(0.453)	–	(0.534)	(7.105)	(0.628)	–	(0.737)	
	^***^		^*^	−	^*^	^***^	^***^	−	^***^	
pI †	114.235	−0.755	0.826	1.456	−	38.394	2.402	1.608	−	0.291
	(5.790)	(0.386)	(0.472)	(0.493)	−	(7.676)	(0.670)	(0.718)	−	
	^***^				–	^**^	^***^	^*^	–	

† *Variable is square-root transformed. Ic, Intercept*.

*Sibs, Total number of siblings ever born*.

*Ef, Father's education. Em, Mother's education. pI, Parental income*.

*SEf, Interaction sex and father's education*.

*SpI, Interaction sex and parental income*.

*SEm, Interaction sex and father's education*.

* p < 0.05;

** p < 0.01;

*** *p < 0.001*.

### 3.3. Model educational attainment

*Father's SEI*, an alternative proxy for parental status, significantly influences children's educational attainment. Similar to our income model, the effect size is strongly influenced by child's sex. The interaction effect of *sex* and *father's SEI* shows that on average sons of high SEI fathers achieve a higher educational attainment than the daughters. In contrast to our income model the *number of siblings* has a significant effect on the respondent's educational attainment. Respondents with a higher number of siblings are less likely to obtain more education on average than their counterparts. This model explains 12% of the observed variance of respondent's educational attainment (see Table 4).

**Table 4:** Effect of father's SEI on respondent's educational attainment.

**Variable**	**Est. of Coefficient**	**Std. Error**	***t*-value**	***p*-value**	
Intercept	12.975	0.074	176.116	<0.001	^***^
No. of siblings	−0.109	0.011	−10.185	<0.001	^***^
Father's SEI	0.022	0.001	13.554	<0.001	^***^
Sex (male = 1)	0.464	0.089	5.187	<0.001	^***^
Sex^*^Father's SEI	0.009	0.002	3.893	0.003	^**^

** p < 0.01;

*** *p < 0.001*.

### 3.4. Model parental ability to support college

The negative effect of *sex* shows that sons of low SEI fathers with no siblings receive less financial support in college than daughters of low SEI fathers with no siblings. The positive coefficient of father's SEI shows that daughters of high SEI fathers with no siblings receive more financial support in college than daughters of low SEI fathers with no siblings. The positive interaction between *sex* and *father's SEI* shows that on average sons of high SEI fathers with no siblings receive more financial support in college than the daughters of high SEI fathers with no siblings (see Table 5).

**Table 5:** Effects of father's SEI on parental ability to financially support college.

**Variable**	**Est. of Coefficient**	**Std. Error**	***t*−value**	***p*−value**	
No. of siblings	−0.179	0.018	−10.119	<0.001	^***^
Father's SEI	0.020	0.002	10.905	<0.001	^***^
Sex (male = 1)	−0.336	0.136	−2.473	<0.001	^***^
Sex^*^Father's SEI	0.003	0.002	1.207	<0.001	^***^
Sex^*^No. of siblings	0.013	0.025	0.518	<0.001	^***^
Intercept 1	−1.023	0.098	−10.400	<0.001	^***^
Intercept 2	1.818	0.102	17.737	<0.001	^***^

*** *p < 0.001*.

## 4. Discussion

In this study we have quantified the effects of parental socioeconomic status—measured by parental income and education—on the next generation's income. Using the WLS data set we have demonstrated that both parental income and parental education contribute strongly to their sons' income and to a lesser degree to their daughters' income. This finding is consistent with the T-W hypothesis, but might as well arise from the difference in opportunities for men and women at this time period independent of differential parental investment. Firstly, the sample of high school students itself may be biased since parents may choose to send only one of their children to college due to financial reasons. Secondly, career opportunities differed for the women and men represented in this sample.

The observed sex differences in income transmission are consistent with Solon (1992) and Mayer and Lopoo (2005), who showed that the intergenerational income elasticity for sons varies between 0.115 and 0.662. In comparison to these findings, the intergenerational income elasticity for daughters varies between 0.39 and 0.41 (Minicozzi, 1997; Shea, 2000; Chadwick and Solon, 2002). However, most of these studies have either been focused on the elasticity for sons or have not taken parental education as well as the number of siblings into account. We demonstrated that parental education, namely father's education, seems to have a stronger indirect influence on son's income than on daughter's income. In contrast to the literature, we found that the number of siblings does not affect the respondent's income significantly in our sample. The intergenerational transmission of income between parents and sons in our sample is likely strong enough to affect the sons' reproductive success. This would be in line with Fieder and Huber (2007, 2012), Nettle and Pollet (2008) and Hopcroft (2006, 2015) that have shown that men's income is positively related to reproductive success, whereas women's income negatively affects the number of their offspring.

The observed sex differences in educational attainment are in line with Hopcroft and Martin (2014), who showed that sons of high SEI fathers had a higher educational attainment than daughters, whereas daughters of low SEI fathers had a higher educational attainment than sons. Furthermore, the result showed that high SEI father's are more likely to be able to financially support their daughters in college than low SEI fathers. Some but not all of our findings are consistent with the T-W hypothesis and with Hopcroft and Martin (2016) whose study showed that sons of high SEI fathers were more likely to receive higher financial parental investment than daughters.

Previous studies tackled the T-W hypothesis with a number of measures of parental investment such as the months of breastfeeding (Gaulin and Robbins, 1991), the time spent with the child (Betzig and Turke, 1986), and how well they know their children's friends (Freese and Powell, 1999). Researchers agreed that parental support for educational attainment is an effective and costly form of investment and thus lends itself to investigating the T-W effect. In view of this, it is surprising that only 48% of studies report a T-W effect in modern human societies (Lazarus, 2002). Two main interpretations emerged why the T-W effect remains elusive. Cronk (2007) argued that in order to unequivocally prove a T-W effect, all acts relying on conscientious forethought on the parts of the parents such as planning and supporting the education of offspring must be controlled for the extent to which economic prospects of the child are determined by sex, since a more basic model of cost vs. payoff may produce a better model of sex differences in attainment of social status, wealth and reproductive success. Hopcroft (2005) cautions that contemporary western societies experience an abundance of resources that leads to high investment in all children. In the case of the WLS sample, both the above mentioned considerations apply: Since all parents could afford to send at least one child to college, they already represent an above average income sample in which the pressure to distribute resources unequally may be low. In addition, parental ability to support college might has been affected by different earning expectations based on the economic situation for daughters and sons of parents coming from different income and social classes.

The main limitations to our study concern the sample. The Wisconsin Longitudinal sample represents mostly white highly educated Americans born between 1937 and 1940. Since the transfer of wealth and status between generations depends on economic and cultural circumstances, our inferences cannot be extended to more modern or culturally different populations. A higher proportion of working women would likely lead to a stronger transfer of income from parents to daughters. Another difficulty lies in the interpretation of the estimate of the coefficients, as income is dependent on too many partially correlated factors to allow a model close to the true model—as evidenced by the proportion of variance explained by our model, 29%. In consequence, the estimates of the coefficients of correlated factors such as parental education and parental income are associated with a high degree of uncertainty. However, repeating the analysis while omitting one factor at a time reveals no major changes in which factors are statistically significant with the notable exception of the factor “mother's education”: The estimated coefficient gains statistical significance when the mother's education is omitted as factors. In addition, variables reliably representing parental investment are difficult to find in historical datasets. While the parental support of higher education represents such an investment, it by no means covers all possible ways in which parents can invest in their offspring.

In conclusion, our results demonstrate an important heritable component of socioeconomic status between parents and sons, and to a lesser extent between parents and daughters. Both parental income and education increase a son's income and educational attainment significantly. In comparison, daughters profit little in terms of income, likely because they face a trade-off between childcare and career outcomes. The results of the present study are consistent with the T-W hypothesis. Future research is needed, however, to clarify whether the observed effects are driven by differential parental investment depending on parental status or solely the product of sociological factors and reproductive trade-offs in later life.

## Author contributions

KEP contributed to the research design/conception, data analyses and writing of the manuscript. AS contributed to data analyses and writing of the manuscript. MF contributed and commented on the research design, writing of the manuscript, and contributed to data analysis.

### Conflict of interest statement

The authors declare that the research was conducted in the absence of any commercial or financial relationships that could be construed as a potential conflict of interest.
